# Elevated periprostatic venous testosterone correlates with prostate cancer progression after radical prostatectomy

**DOI:** 10.1172/JCI171117

**Published:** 2023-09-01

**Authors:** Mohammad Alyamani, Patrick Michael, Daniel Hettel, Lewis Thomas, Scott D. Lundy, Mike Berk, Mona Patel, Jianbo Li, Hooman Rashidi, Jesse K. McKenney, Eric A. Klein, Nima Sharifi

**Affiliations:** 1 Genitourinary Malignancies Research Center, Lerner Research Institute,; 2 Department of Urology, Glickman Urological and Kidney Institute,; 3 Department of Quantitative Health Sciences, Lerner Research Institute,; 4 Department of Pathology, Pathology and Laboratory Medicine Institute, and; 5 Department of Hematology and Oncology, Taussig Cancer Institute, Cleveland Clinic, Cleveland, Ohio, USA.

**Keywords:** Endocrinology, Oncology, Prostate cancer, Sex hormones, Urology

## Abstract

**BACKGROUND:**

Generally, clinical assessment of gonadal testosterone (T) in human physiology is determined using concentrations measured in peripheral blood. Prostatic T exposure is similarly thought to be determined from peripheral T exposure. Despite the fact that androgens drive prostate cancer, peripheral T has had no role in the clinical evaluation or treatment of men with localized prostate cancer.

**METHODS:**

To assess the role of local androgen delivery in prostate cancer, we obtained blood from the (periprostatic) prostatic dorsal venous complex in 266 men undergoing radical prostatectomy from July 2014 to August 2021 and compared dorsal T (DT) levels with those in circulating peripheral blood (PT) and prostatic tissue. Comprehensive targeted steroid analysis and unbiased metabolomics analyses were performed. The association between the DT/PT ratio and progression-free survival after prostatectomy was assessed.

**RESULTS:**

Surprisingly, in some men, DT levels were enriched several-fold compared with PT levels. For example, 20% of men had local T concentrations that were at least 2-fold higher than peripheral T concentrations. Isocaproic acid, a byproduct of androgen biosynthesis, and 17-OH-progesterone, a marker of intratesticular T, were also enriched in the dorsal vein of these men, consistent with testicular shunting. Men with enriched DT had higher rates of prostate cancer recurrence. DT/PT concentration ratios predicted worse outcomes even when accounting for known clinical predictors.

**CONCLUSIONS:**

These data suggest that a large proportion of men have a previously unappreciated exposure to an undiluted and highly concentrated T supply. Elevated periprostatic T exposure was associated with worse clinical outcomes after radical prostatectomy.

**FUNDING:**

National Cancer Institute (NCI), NIH grants R01CA172382, R01CA236780, R01CA261995, R01CA249279, and R50CA251961; US Army Medical Research and Development Command grants W81XWH2010137 and W81XWH-22-1-0082.

## Graphical abstract

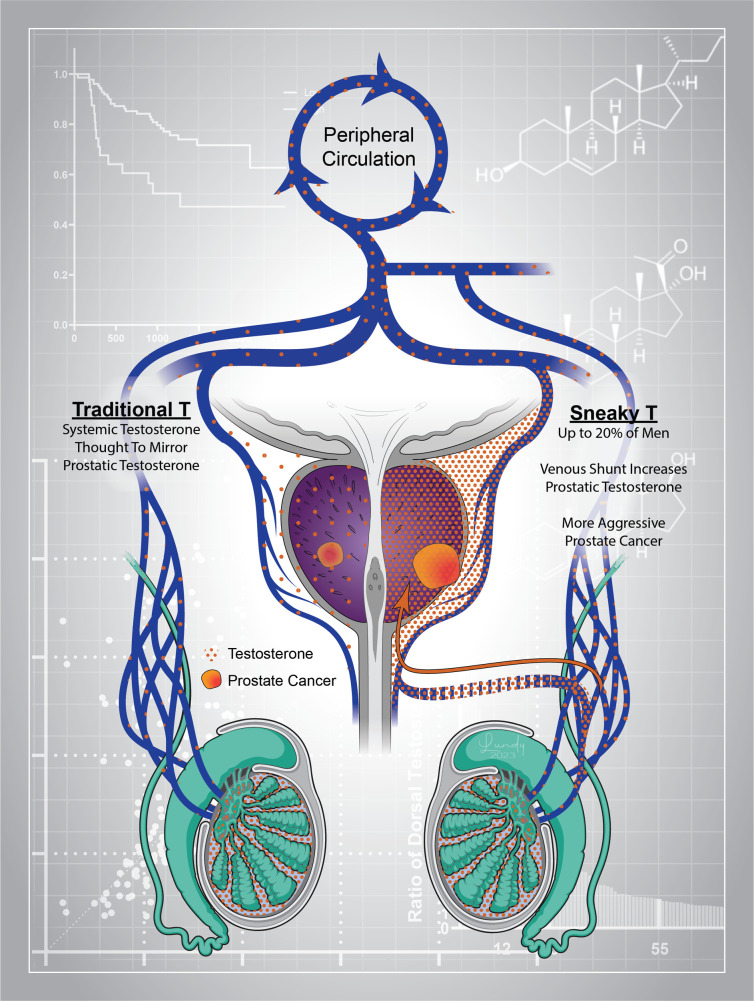

## Introduction

Androgens and androgen receptor (AR) signaling are essential for normal prostate development, the initiation of prostate cancer, and tumor progression even at very advanced stages (1). Gonadal testosterone (T), the predominant androgen in circulation, is the major source of prostatic androgen exposure. In the prostate, T is converted by 5α-reductase isoenzymes to 5α-dihydrotestosterone (DHT), both of which stimulate the AR (2, 3). Traditional anatomic and physiologic dogma states that the gonadal veins carry freshly synthesized T at high concentrations from the left testicle to the left renal vein, or from the right testicle to the inferior vena cava, where it is rapidly diluted by 2 orders of magnitude within the systemic circulation (4) before delivery at a much lower and presumably physiologic concentration to peripheral tissues including the prostate (5, 6). All studies that interrogate the effect of T on the prostate — whether they be studies of prostate cancer risk (7, 8), prostate cancer clinical response to medical castration (9), the occurrence of T flare that transpires with some forms of hormonal therapy (10), or any other physiologic or clinical outcome — are based on the underlying assumption that peripheral circulation, which is easily and simply assessed by upper extremity venipuncture (11), accurately represents the hormonal milieu experienced by the prostate. Furthermore, peripheral T measurements are not routinely used for the clinical management of men who undergo radical prostatectomy for localized prostate cancer.

Here, in the first intraoperative study of its kind to our knowledge, we investigated androgen physiology in 266 men with prostate cancer undergoing open radical prostatectomy between 2014 and 2021. Three physiologic compartments were collected simultaneously in patients and assessed: (a) intraoperatively obtained dorsal vein blood, (b) peripheral blood, and (c) fresh prostatic tissue. Dorsal vein blood was obtained by direct cannulation of the superficial branch of the prostatic venous plexus (also known as Santorini’s plexus), a network of richly anastomosing veins that comprise the primary venous drainage of the prostate. We performed comprehensive targeted steroid mass spectrometry, unbiased metabolomics from outlier patients, and comprehensive clinical follow-up. Surprisingly, our study shows that some men have significantly enriched periprostatic venous T levels accompanied by biochemical markers of an intratesticular source, including specific steroidal intermediates and the side chain cleavage byproduct that marks the first enzymatic step for T synthesis in the Leydig cells of the testes. These men had worse clinical outcomes after surgery even when accounting for the usual clinical predictors. Together, these data suggest that, in a subset of men, there exists an alternative local androgen physiology we have termed “sneaky” T physiology that exposes the prostate to enriched gonadal T and is associated with poor clinical outcomes in men with prostate cancer.

## Results

**Figure 1:**
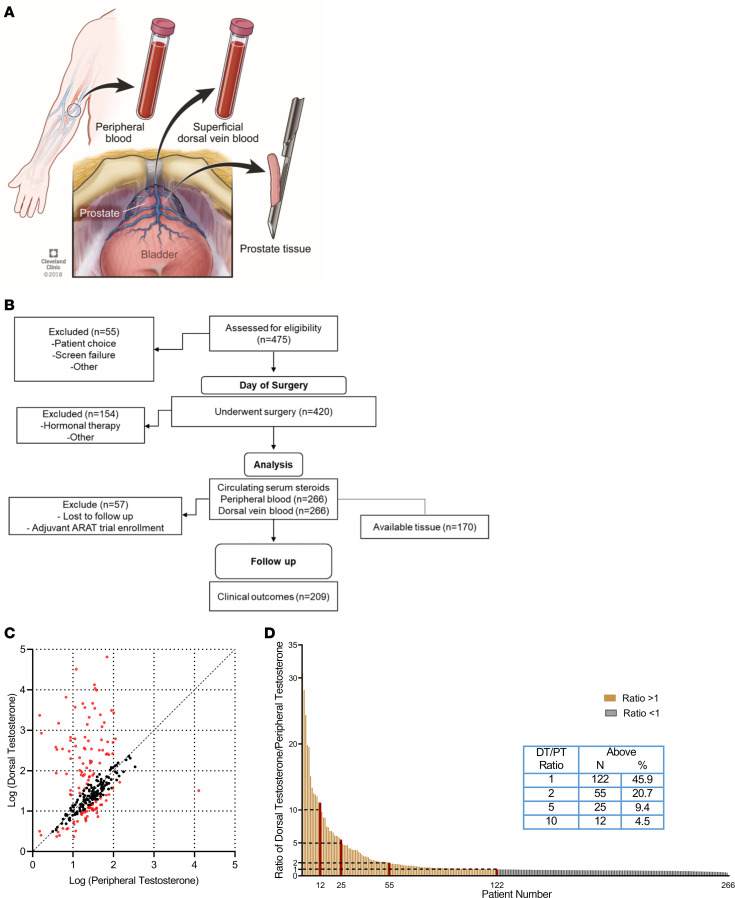
An intraoperative study in men with prostate cancer shows that local T exceeds PT in a subset of men. (**A**) Study schema showing same-day collection of peripheral blood, intraoperative superficial dorsal vein blood, and prostate tissue. (**B**) CONSORT diagram detailing the study inclusion criteria and biospecimen collection. ARAT, androgen receptor axis–targeted therapy. (**C**) DT and PT deviated significantly from the underlying assumption that they would be of the same distribution (Kolmogorov-Smirnov test *P* < 0.001). (**D**) Distribution of the ratio of DT/PT for men in the cohort. Over 20% of the men in the cohort had DT/PT ratios of greater than 2. Red bars indicate the lowest individual value that exceeded the thresholds of a DT/PT ratio of greater than 1, 2, 5, or 10.

The study design included the collection of peripheral blood, dorsal vein blood, and prostatic tissue at the time of radical prostatectomy (Figure 1A). Of the 475 men who were recruited for the study, 420 subsequently underwent radical prostatectomy. Of these, 266 patients with no prior hormonal therapy had adequate samples collected for analysis. We then performed steroid analysis of peripheral and dorsal vein blood. Tissue samples from 170 prostates were analyzed, and data were available for assessment of clinical outcomes for 209 men (Figure 1B and Supplemental Table 1; supplemental material available online with this article; https://doi.org/10.1172/JCI171117DS1). Remarkably, the distribution of dorsal vein testosterone (DT) and peripheral testosterone (PT) in the study participants deviated from the assumption of classical physiology that they would be of the same or similar distribution (Figure 1C). Instead, the distribution was highly skewed toward elevated DT (*P* < 0.001 using the Kolmogorov-Smirnov test). In fact, the ratio of T in dorsal vein to T in peripheral blood (DT/PT) in individuals demonstrates that a substantial proportion of the men with prostate cancer had periprostatic T levels that far exceeded the corresponding levels in the peripheral circulation. At various DT/PT cut points, these ratios were largely driven by the substantial variations in DT (Supplemental Table 2). Approximately 20% of men had local T concentrations at least 2-fold higher than peripheral T, and approximately 5% of men had at least a 10-fold elevation of periprostatic T levels (Figure 1D). Together, these data are incompatible with the general assumption that prostatic androgen exposure can be estimated by measuring peripheral circulating T levels and suggest that a subset of men experience unrecognized vigorous androgen stimulation.

**Figure 2:**
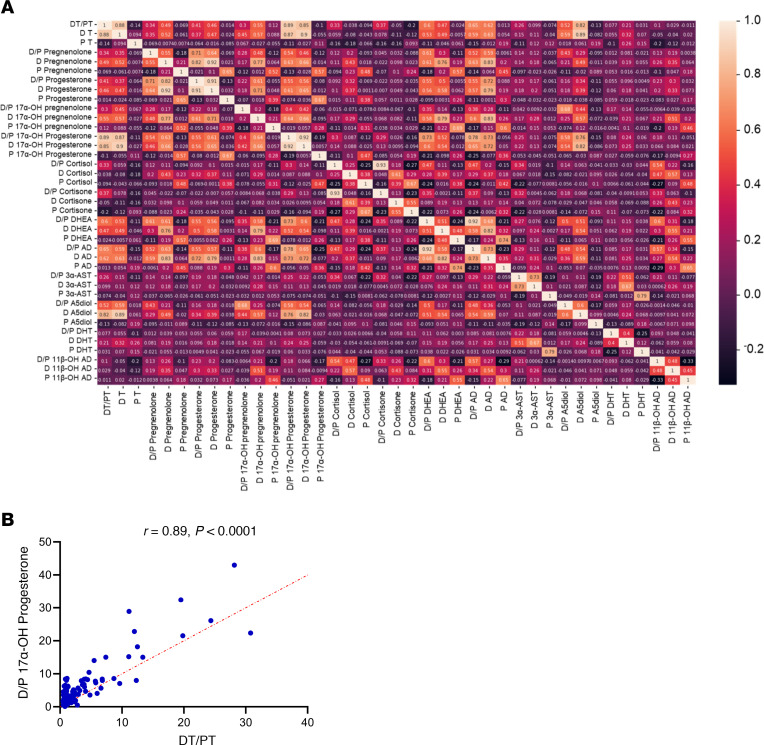
Comprehensive targeted mass spectrometry steroid profiling identifies 17-OH-progesterone and suggests intratesticular origins of “sneaky” T. (**A**) Of the 13 steroids tested, 17-OH-progesterone was most strongly associated with T ratios in the superficial dorsal vein compared with peripheral blood. (**B**) Pearson’s correlation analysis between T and 17-OH-progesterone ratios in dorsal compared with peripheral serum.

To further investigate the mechanism of local periprostatic T enrichment, peripheral blood and dorsal vein blood were comprehensively profiled for steroids using stable isotope dilution targeted mass spectrometry to definitively identify steroids co-associated with T and identify the anatomic and biochemical origins in individuals with elevated DT. We profiled 13 total steroids in both the dorsal and peripheral blood samples. For each steroid, correlations with absolute concentrations and the ratio of dorsal vein/peripheral blood were determined. Of these, the dorsal vein/peripheral blood ratio for 17-OH-progesterone was most strongly associated with DT/PT (*r* = 0.89; *P* < 0.0001) (Figure 2, A and B). 17-OH-progesterone is established as a serum marker that strongly correlates with intratesticular T production and is used for evaluation and treatment monitoring in men with infertility (12, 13). These data therefore suggest that the enriched periprostatic androgens are testicular in origin.

**Figure 3:**
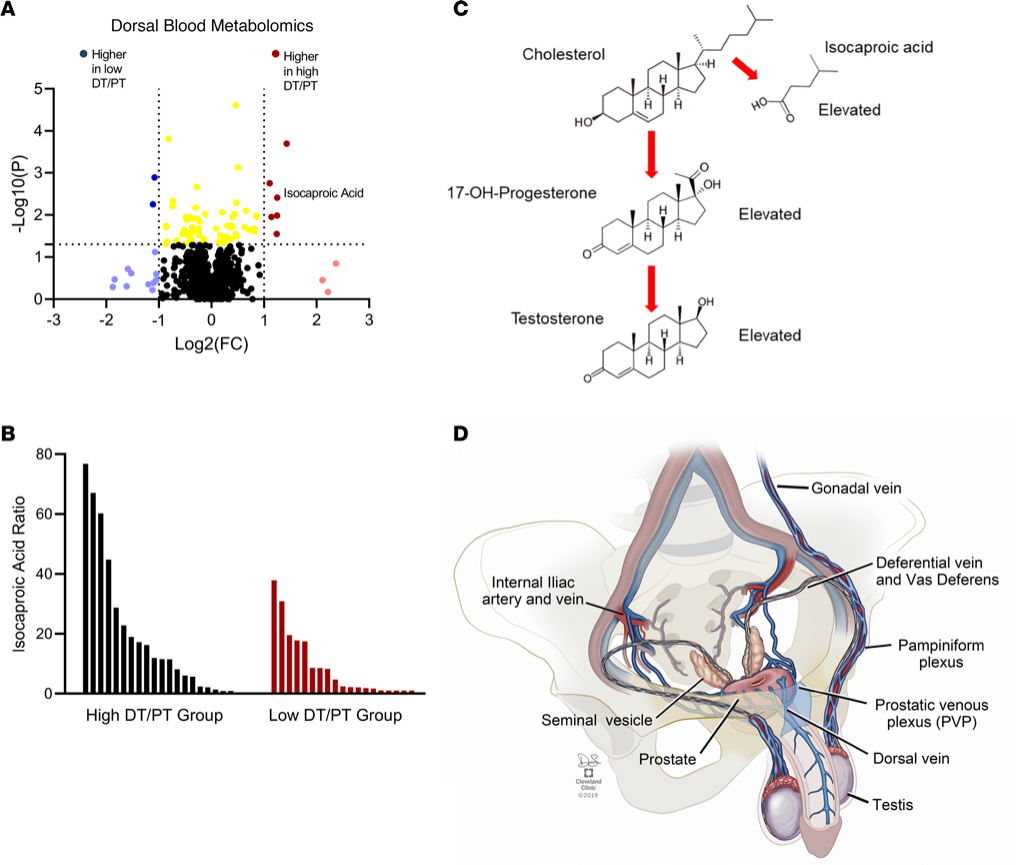
Untargeted metabolomics profiling of men with DT/PT outliers identifies the byproduct of the first enzymatic step of de novo T biosynthesis. (**A**) Unbiased metabolomics analysis of dorsal vein blood in men with DT/PT outliers yielded isocaproic acid (fold change [FC] = 2.38; *P* = 0.004) as a top distinguishing metabolite and is indicative of a source of androgen biosynthesis. (**B**) Comparison of dorsal to peripheral blood isocaproic acid ratios in men with high DT/PT versus low DT/PT ratios. (**C**) Simplified schema of androgen biosynthesis in the Leydig cells of the testes. The first enzymatic step requires side chain cleavage and results in the 6-carbon byproduct isocaproic acid. 17-OH-progesterone is an intermediate steroid metabolite that is a validated marker of intratesticular T. (**D**) One of several plausible routes of vascular shunting that enables testicular drainage via the deferential vein to the vasculature abutting the prostate.

To further interrogate the metabolic perturbations that occur in individuals with elevated DT/PT ratios, we investigated dorsal and peripheral sera using unbiased metabolomics in the 40 men with outlier DT/PT values (20 high and 20 low). Isocaproic acid was among the top metabolites that was specifically elevated in the dorsal vein in men with a high compared with low DT/PT ratio (Figure 3A and Supplemental Figure 1). Furthermore, the ratio of isocaproic acid in dorsal compared with peripheral serum was similarly higher in men with a high DT/PT value compared with the low DT/PT group (Figure 3B). Isocaproic acid is the 6-carbon side chain cleavage product from carbons 22 to 27 of cholesterol that results from CYP11A1-mediated enzymatic catalysis of the first step of T biosynthesis in the testicular Leydig cells (Figure 3C). Along with the elevated 17-OH-progesterone concentration, the higher concentration of isocaproic acid that accompanies increased periprostatic T in men with an elevated DT/PT ratio is further direct biochemical evidence of enrichment occurring directly from gonadal T biosynthesis.

Testicular venous drainage involves multiple small collaterals including the gonadal, cremasteric, and deferential venous plexi (14). In a subset of men, this network includes a direct anastomosis to the prostatic venous plexus and provides an anatomic basis for the hypothesized mechanism of elevated periprostatic T (Figure 3D). Interestingly, prior work has shown that men with bilateral varicocele, defined as the presence of dilated spermatic veins with sluggish flow, exhibited an increased dorsal venous complex diameter (15).

Manipulation of T in the circulation affects intraprostatic androgens. However, the androgen changes in the prostate that follow do not always clearly reflect changes in circulating T (16–18). Previously unappreciated prostatic androgen exposure that is unassessable when only peripheral T is measured with traditional phlebotomy is a possible contributing explanation. As such, we next wished to determine whether the men with elevated periprostatic T had evidence for increased androgen exposure in the prostate above what would be expected using peripherally obtained T values. In other words, does the subset of men with higher-than-expected periprostatic T also have higher prostate tissue androgens when normalized to peripheral T? T, DHT, and the major downstream inactive androgen androsterone (AST) were assessed by mass spectrometry using fresh-frozen prostate tissue obtained from radical prostatectomy for the 170 patients for whom tissues were available. After normalization to peripheral T levels, we compared the levels of T, DHT, and AST in men with elevated periprostatic T (DT/PT >1) versus those without elevated levels. Recognizing the potential for variability in blood flow to the prostate, we suspected that any specific contribution of elevated T from the periprostatic venous system would be apparent in men with the higher observed prostate tissue T levels. We therefore compared prostate tissue T above the median (i.e., top 2 quartiles) for those in the high periprostatic T group with those in the low periprostatic T group (Supplemental Figure 2A). We found that normalized prostate tissue T levels were significantly greater in men with high periprostatic T compared with those who had low periprostatic T levels (*P* < 0.001). Thus, our data suggest that when this variant physiology is present, the prostate is indeed exposed and that the upper range of prostate tissue T exposure increases with higher concentrations of augmented periprostatic T. We also assessed prostate tissue DHT and similarly observed that normalized tissue DHT was significantly higher in the elevated periprostatic T group compared to the low periprostatic T group (*P* = 0.004; Supplemental Figure 2B). Significant increases in prostate tissue AST, the major inactive 5α-reduced androgen downstream of DHT, were similarly present in the elevated periprostatic T group (*P* = 0.02; Supplemental Figure 2C). We also performed the same analyses across various DT/PT ratio cut points from 1 to 3 (Supplemental Table 3). These analyses show that elevated prostate tissue T (all cut points), DHT, and AST (most cut points) generally occurred in the high DT group across a range of DT/PT cut points. Together, these data suggest that the augmented gonadal T in the periprostatic venous vasculature penetrated prostatic tissue, was converted by intraprostatic 5α-reductase to DHT, and was then inactivated to the major inactive androgen AST.

**Figure 4:**
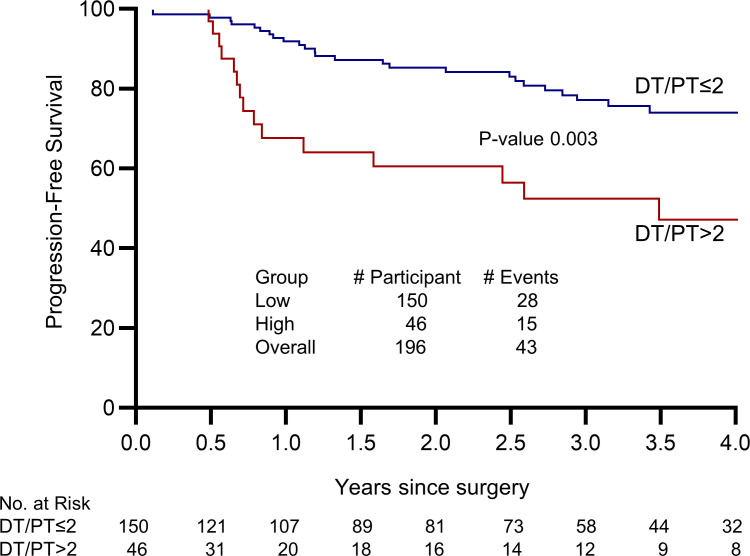
Elevated DT/PT ratio (>2) is associated with adverse clinical outcomes in men after radical prostatectomy. Progression-free survival was defined using PSA recurrence or radiation therapy as clinical events. Included are men (*n* = 196) without PSA persistence after radical prostatectomy.

Finally, we determined the association between clinical outcomes in men with elevated DT/PT using a ratio of 2 as a cut point. We assessed the time from surgery to a composite clinical event defined by the first occurrence of either prostate-specific antigen (PSA) recurrence using National Comprehensive Cancer Network criteria or initiation of radiation therapy. PSA persistence frequently occurs in men whose disease has metastasized. These sites of micrometastases may not therefore be exposed to elevated periprostatic androgens. For example, 7 of 13 men with PSA persistence in this cohort had evidence of lymph node involvement on final pathology. Tumor cells that seed the lymph nodes may not be exposed to the same effects of a high DT/PT ratio that occurs in the prostate itself, and progression may no longer be within the context of the elevated periprostatic venous T levels. Men with a DT/PT ratio of greater than 2 had a higher rate of clinical events compared with those who had a ratio of less than 2 (Figure 4; *P* = 0.003). Analyses including men with PSA persistence similarly showed poorer outcomes for men with a DT/PT of greater than 2 (Supplemental Figure 3; *P* = 0.034). We further sought to determine whether the prognostic information garnered from knowing the DT/PT physiology is independent of known clinical variables and how it functions across a range of DT/PT cut points. Multivariable analyses incorporating preoperative PSA, extraprostatic extension (pathologic stage pT3), prostatectomy Gleason score, age, and race showed an even stronger association between DT/PT and clinical outcomes (Tables 1 and 2). Notably, this relationship held true across a range of DT/PT cut points, whether all 209 men were included (Table 2) or those with PSA persistence were excluded (Table 1). Together, these data suggest that an unexpected direct androgen prostatic delivery pathway exists in a subset of men with prostate cancer and is associated with adverse clinical outcomes after prostatectomy independent of other known prognostic variables.

## Discussion

In this study, we performed direct biochemical assessment of 3 physiologic compartments in a large series of men with prostate cancer undergoing radical prostatectomy and identified a previously unrecognized locally concentrated endocrine phenomenon that was associated with worse clinical outcomes. To our knowledge, this is the first study providing evidence that periprostatic venous blood is significantly enriched for T in some men and has direct physiologic effects on the prostate. The identification of both a steroid intermediate and a nonsteroid byproduct of T biosynthesis that track with elevated periprostatic T suggests that enrichment indeed arises from the primary source of T biosynthesis, the testicular Leydig cells. Although other origins of steroidogenesis are possible, the tracking of dorsal/peripheral ratios of T with 17-OH-progesterone, a well-established marker of gonadal function, supports testicular steroidogenesis as the most probable source (13, 19).

We considered the possibility that our findings are confounded by the timing of blood collection, i.e., peripheral blood collection prior to the induction of anesthesia and collection of the blood from the superficial dorsal vein during surgery. However, clinical evidence suggests that, compared with preoperative levels, PT levels may decrease, not increase, with surgery (20). Therefore, it seems highly unlikely that the elevated periprostatic T is attributable to the timing of blood collection.

Although the testicular source of enriched T in periprostatic blood is well supported by our data, the mechanism by which this occurs is speculative. A direct route of venous shunting, wherein gonadal T bypasses the systemic circulation by flowing directly from the testes to the prostate, has been theorized (21). This mechanism remains largely unproven but could account in part for the pathogenesis of androgen-dependent prostatic pathology. We provide evidence to support extension of this hypothesis to suggest that development of this venous shunt may occur from a shift in internal spermatic vein (ISV) blood flow toward collateral pathways involving the prostatic venous drainage network. Interestingly, in a small study of 6 men, gonadal vein ablation was reported to lead to declines in prostate volume and PSA in a proportion of cases (21). Varicocele, a clinical manifestation of impaired ISV valve function causing poor venous outflow, congestion, and hyperthermia, is associated with significantly decreased circulating T levels (22). The lower peripheral T concentrations in the men with high DT/PT ratios in our study (Supplemental Table 2) are compatible with compromised gonadal function and venous drainage and lend support to the hypothesis that periprostatic T enrichment occurs via venous shunting. Varicocele repair improves gonadal function and increases testicular size, improves semen parameters, and increases peripheral serum T levels (23). Varicocele is the most common known cause of infertility, and some evidence suggests that an association may exist between infertility and risk of aggressive prostate cancer (24). However, no plausible mechanism has been postulated to support this association to date. The physiology identified here and the link to elevated periprostatic androgens provides a potential mechanistic link to aggressive prostate cancer and poor clinical outcomes. Further study is warranted to determine whether an association between varicocele and worsened outcomes for men with prostate cancer exists, as this may warrant increased varicocele surveillance and serve as an additional indication for varicocelectomy.

These data suggest that the true degree of prostatic androgen exposure is missed when only peripheral androgens in venous circulation are measured. The potential implications for this finding are broad and profound. In men who already have androgen-dependent prostatic diseases such as benign prostatic hyperplasia or prostate cancer, knowledge of the absence, presence, and extent of this “sneaky” T physiology and total prostatic androgen exposure may further clarify an individual’s distinct natural history of the disease. Notably, the poor outcomes associated with high periprostatic T levels in our study are independent of known clinical factors, including pathology and preoperative PSA. Furthermore, the multivariable modeling shows that the poor outcomes are predicted for high periprostatic venous T levels across a broad range of DT/PT ratio cut points, which increases the robustness of these findings and suggests a continuous effect of periprostatic androgen physiology on clinical outcomes. Hypogonadism, according to traditional peripheral T measurement, represents an interesting related topic whereby men with prostate cancer may have a paradoxical elevated prostatic androgen exposure because of this sneaky T physiology. Similarly, men with sneaky T physiology treated with gonadotropin-releasing hormone analogs for prostate cancer may have much higher prostatic exposure to androgens than would be surmised by assessing the decline in peripheral T levels.

In conclusion, we found that a subset of men with prostate cancer had unexpectedly elevated periprostatic venous T levels and poor clinical outcomes after radical prostatectomy. The identification of men with sneaky T physiology adds new information for the prognostication of clinical outcomes beyond what is currently assessed in clinical practice. More generally, these findings of an alternative local endocrine physiology have broad potential implications for the assessment of risk in diseases of the prostate and implicate potential alternative treatment strategies for urologic diseases that are modulated by sneaky T physiology.

## Methods

### Patient selection.

Patients with localized prostate cancer were recruited from the clinical practice of a urologist at the Cleveland Clinic between July 2014 and August 2021. All participants provided written informed consent before enrolling under a Cleveland Clinic IRB-approved protocol (Case 5923).

Of the 475 patients initially recruited, 420 patients subsequently underwent open radical prostatectomy. Of these, 266 patients had no prior hormonal therapy and had both dorsal and peripheral blood samples collected. Of the 266 patients, 170 had prostate tissue collected.

### Patient serum and tissue collection.

Just prior to the induction of anesthesia, a peripheral venous blood sample was collected from each patient. During surgery, the primary surgeon then collected a blood sample from the superficial branch of the dorsal venous complex by cannulating the branch with a butterfly needle (Figure 1). Blood was collected in red top Vacutainer Plus plastic serum collection tubes (BD367814, Becton Dickinson) and allowed to clot. The samples were centrifuged at 1,430*g* for 10 minutes at room temperature within 1 hour of collection. Serum aliquots were frozen at –80°C until processing for analysis. After prostatectomy, prostate tissue was collected within 15 minutes of prostate removal and kept frozen at –80°C until analysis.

Additional methods related to mass spectrometry (25), statistical, and clinical analyses are available in the Supplemental Methods.

### Statistics.

Results are expressed as the median and IQR or counts and percentages. We used the Mann-Whitney *U* test to compare continuous non-normal variables between groups. For categorical variables, the comparisons were made using the χ^2^ test or Fisher’s exact text. Pearson correlation was used to assess the association between variables. The Kolmogorov-Smirnov test was used to test for common distribution. Kaplan-Meier analysis and a log-rank test were used to compare survival between groups. A Cox proportional hazards model was applied to account for the effect of demographic, clinical, and pathologic variables on survival between groups. All data analyses were conducted using the stat and survival packages in R (www.r-project.org), and results were considered significant at the α = 0.05 level.

### Study approval.

The studies on human samples were reviewed and approved by the IRB at the Cleveland Clinic. All participants provided appropriate prior written informed consent for participation in the study.

### Data and materials availability.

All supporting data related to graphs and tables are available in the Supporting data values file.

## Author contributions

MA performed experiments and analyzed data. PM, DH, LT, JKM, and SDL analyzed clinical data. MB and MP managed clinical biospecimens. JL and HR performed statistical analyses. EAK and NS oversaw the project. NS, MA, PM, and EAK wrote the first draft of the manuscript, and all authors reviewed and edited the manuscript.

## Supplementary Material

Supplemental data

ICMJE disclosure forms

Supporting data values

## Figures and Tables

**Table 1:**
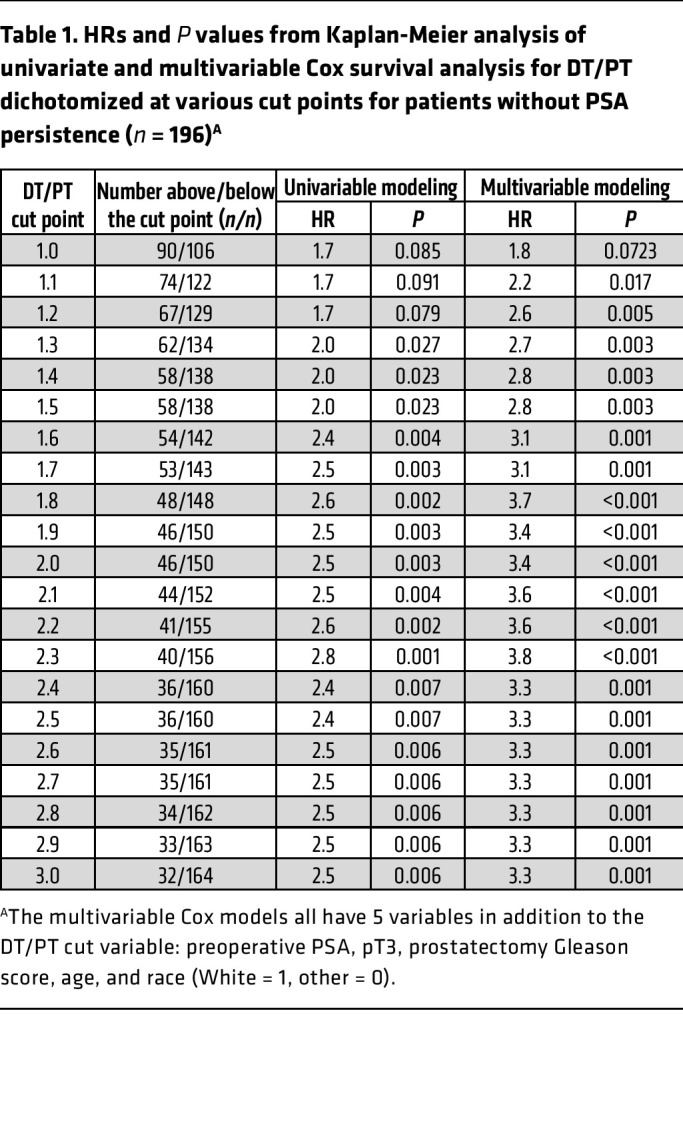
HRs and *P* values from Kaplan-Meier analysis of univariate and multivariable Cox survival analysis for DT/PT dichotomized at various cut points for patients without PSA persistence (*n* = 196)^A^

**Table 2:**
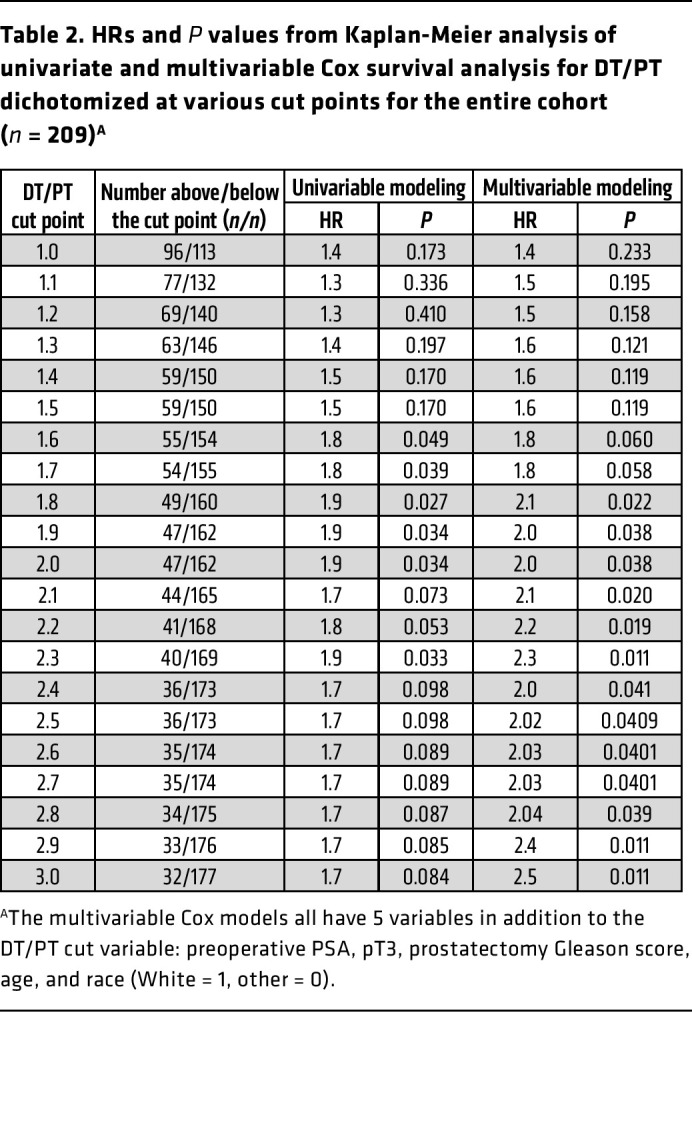
HRs and *P* values from Kaplan-Meier analysis of univariate and multivariable Cox survival analysis for DT/PT dichotomized at various cut points for the entire cohort (*n* = 209)^A^

## Version 1 09/01/2023

Electronic publication

## Version 2 09/06/2023

Fixed 2 grammar errors
