# Supplementary material for: Elevated periprostatic venous testosterone correlates with prostate cancer progression after radical prostatectomy
Source: J Clin Invest. 2023 Sep 1;133(17):e171117. doi: 10.1172/JCI171117 (PMC10471166; doi:10.1172/JCI171117)
Supplement: Supplemental data [file jci-133-171117-s007.pdf]

Supplementary Appendix

Elevated periprostatic venous testosterone correlates with prostate cancer progression after radical prostatectomy

Mohammad Alyamani, PhD[1], Patrick Michael, MD[1,2], Daniel Hettel, MD[1,2], Lewis Thomas, MD[1,2], Scott D. Lundy, MD, PhD[2], Mike Berk, MS[1], Mona Patel, BS[1], Jianbo Li, PhD[4], Hooman Rashidi, MD[5], Jesse K. McKenney, MD[5], Eric A. Klein, MD[1,2], Nima Sharifi, MD[1,2,3,6]

[1]Genitourinary Malignancies Research Center, Lerner Research Institute, Cleveland Clinic, Cleveland, OH, USA

[2]Department of Urology, Glickman Urological and Kidney Institute, Cleveland Clinic, Cleveland, OH, USA

[3]Department of Hematology and Oncology, Taussig Cancer Institute, Cleveland Clinic, Cleveland, OH, USA

[4]Department of Quantitative Health Sciences, Lerner Research Institute, Cleveland Clinic, Cleveland, OH, USA

[5]Department of Pathology, Pathology and Laboratory Medicine Institute, Cleveland Clinic, Cleveland, OH, USA

[*] Correspondence: Nima Sharifi

[6]Desai Sethi Urology Institute, University of Miami Miller School of Medicine, Miami, FL, USA

[*]Correspondence: Nima Sharifi

Phone: 305 243-7014

Email: nxs1664@miami.edu

Materials and Methods

Serum and tissue steroid extraction and LC-MS analysis

Thirteen steroids -- pregnenolone, progesterone, 17α-OH pregnenolone, 17α-OH progesterone, cortisol, cortisone, 11β-OH androstenedione (11β-OH-AD) dehydroepiandrosterone (DHEA), androstenedione (AD), androstanediol (A5diol), testosterone (T), 5α-dihydrotestosterone (DHT), 3α-androsterone (AST) -- and the spiked internal standards pregnenolone-d4, progesterone-d9, DHEA-d2, $^{3}C_{13}$-AD, A5diol-d3, T-d3,DHT-d3, and AST-d2 (all from Steraloids) were extracted from patient serum according to our previously published liquid-liquid extraction protocol (24); the steroids were derivatized with hydroxylamine (Fluka) (0.75 µM) and extracted with 2 mL methyl tert-butyl ether (ACROS), and were separated by reversed phase chromatography.

To extract T, DHT, and AST from the prostate tissue, 5 to 25 mg tissue was homogenized with 500 µL methanol (Fisher) containing the internal standard (5 ng/ml of T-d3, DHT-d3, and AST-d2). The androgens were extracted from the homogenate as follows: after derivatization with 0.75 µM hydroxylamine, the samples were loaded onto solid-phase cartridges that were conditioned with 1 mL methanol and 1 mL water; then, after washing with water, the analytes were eluted with 1 mL methanol. The samples were evaporated and reconstituted with methanol:water, 50:50.

The LC-MS/MS system was an ultra-pressure liquid chromatography system (Shimadzu Corporation, Japan) consisting of two LC-30AD pumps, a DGU-20A5R degasser, a CTO-30A column oven, SIL-30AC autosampler, and a system controller CBM-20A coupled with a Qtrap 5500 mass spectrometer (AB Sciex). Data acquisition and processing were performed using Analyst software (version 1.6.2) from ABSciex.

Steroids were ionized using electrospray ionization in positive mode. Analytes were quantified using multiple reaction monitoring. The steroids and their internal standards were separated using a Zorbax Eclipse plus C18 column (Agilent) with a mobile phase consisting of (A) 0.1% formic acid in water:methanol, 70:30 and (B) 0.1% formic acid in acetonitrile:methanol:water, 50:30:20, with a gradient program at a flow rate of 0.2 mL/min. Sample injection volume was 10 µL.

Steroid Analysis

The multicollinearity assessment App within the suite of MILO's (Machine Intelligence Learning Optimizer) automated machine learning and statistical software tools, was used to generate and assess the initial correlations between the steroid concentrations in peripheral blood, dorsal blood, as well as the ratio of peripheral to dorsal blood samples for each steroid profiled.

The correlation heat map shown is a graphical representation of the correlations between the different variables in this dataset. Each square on the grid represents the relationship between two variables. The color of each square indicates the strength of the relationship, with darker colors indicating a stronger relationship. The r value is the Pearson correlation coefficient.

Untargeted metabolomics analysis

Peripheral and dorsal samples from 40 patients (20 with DT/PT ratio < 0.87 and 20 with DT/PT ratio > 4.50) were subject to untargeted metabolomics. The analysis was carried out by Metabolon, Inc., (https://www.metabolon.com). In brief, samples were prepared using the automated MicroLab STAR system (Hamilton Company). Proteins were precipitated with methanol followed by centrifugation. The resulting extract was divided into five fractions: two for analysis by two

separate reverse phase (RP)/UPLC-MS/MS methods with positive ion mode electrospray ionization (ESI), one for analysis by RP/UPLC-MS/MS with negative ion mode ESI, one for analysis by HILIC/UPLC-MS/MS with negative ion mode ESI, and one sample was reserved for backup. Samples were placed briefly on a TurboVap (Zymark) to remove the organic solvent. The sample extracts were stored overnight under nitrogen before preparation for analysis. Data analysis was performed using MetaboAnalyst (V5.0). Any feature with more than 50% missing values was excluded from the analysis, and missing values were replaced by 1/5 of the minimum value. Data were normalized to the median, log transformed, and auto-scaled. Data were assumed to be nonparametric, and significance of the difference between the groups was tested using the Mann Whitney U test.

Clinical Analysis

For the clinical outcomes analysis, we performed a chart review of the 266 patients with both peripheral and dorsal vein blood samples. Of these patients, 57 were excluded because they enrolled after October 2019, which would not have allowed for adequate follow up time to establish clinical outcomes, lack of post-operative follow-up, or enrollment in a separate trial of adjuvant enzalutamide. The final cohort of 209 patients was used for analysis of clinical outcomes.

The clinical endpoint for this study was biochemical recurrence as defined by the National Comprehensive Cancer Network (NCCN) criteria: an undetectable PSA level after prostatectomy with a subsequent detectable PSA level that increases on 2 or more determinations, or progression to adjuvant radiation therapy (XRT) due to a high-risk Decipher score. Our patients were recommended for adjuvant XRT due to an intermediate- or high-risk Decipher score based on evaluation of the prostatectomy specimen. The Genomic Decipher Score is a prostate cancer gene-expression classifier that predicts an individual patient’s risk of metastasis and prostate cancer-

specific mortality. We decided to include progression to adjuvant XRT due to the Decipher score value as a part of the clinical endpoint because radiation therapy limits the evaluation of those patients for biochemical recurrence.

Statistical Analysis

We used the Kolmogorov-Smirnov test for common distribution to test the assumption that peripheral testosterone and dorsal testosterone are the same concentration. We divided our cohort into two groups based on the dorsal testosterone / peripheral testosterone ratio: DT/PT >1 (n= 122) and DT/PT ≤1 (n=144). Tissue androgens were compared between the two groups using the Mann-Whitney U test. Given that the PT and DT distributions differed the most above their medians, comparisons were also made between the top two quartiles of the data using the Mann-Whitney U test. Where necessary, data were transformed only for clear visualization. All data analyses were done using the statistical software R (www.r-project.org), and results were considered significant at the $\alpha=0.05$ level.

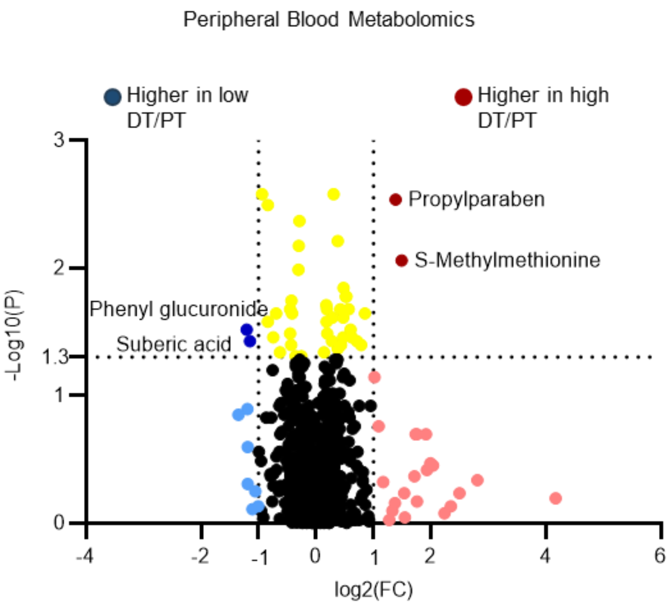

Fig. S1. Peripheral blood metabolomics comparison between men who have high vs. low DT/PT. Unlike the comparison between the dorsal vein blood in the same men, isocaproic acid is not among the metabolites that differ significantly between the groups.

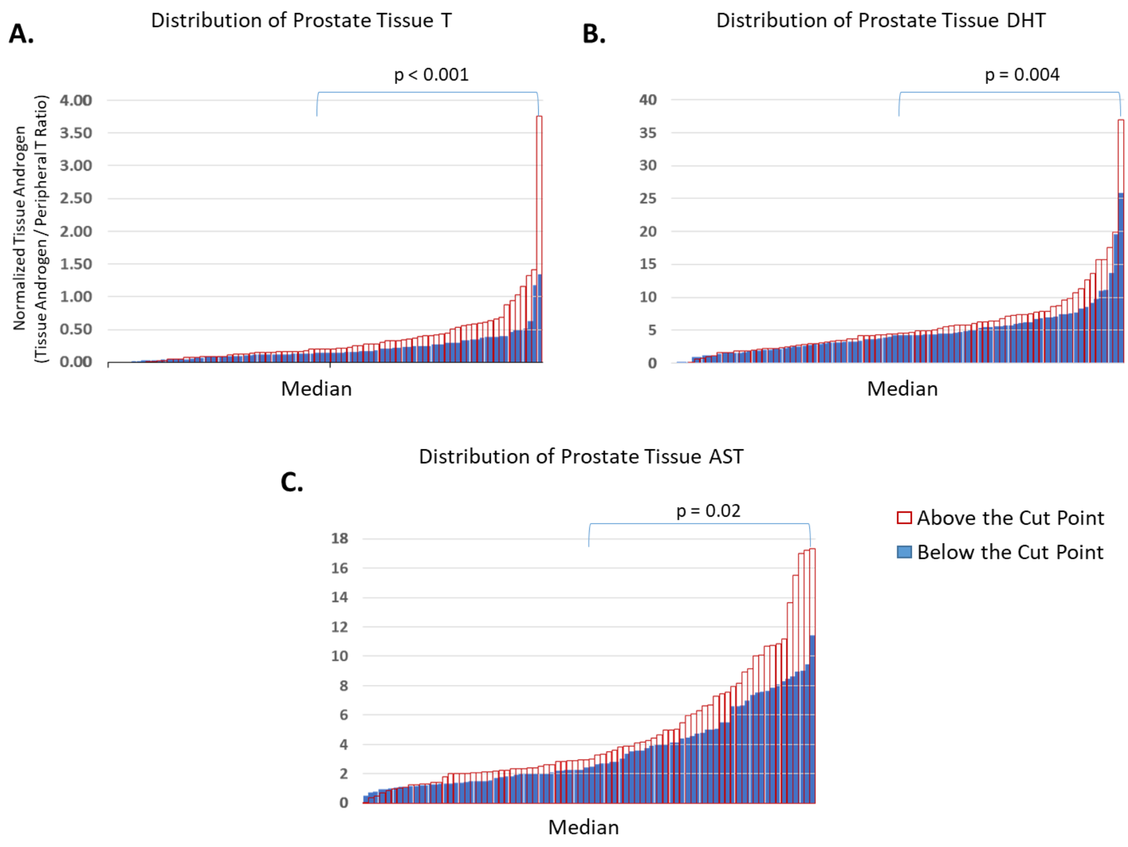

Fig. S2. Elevated dorsal vein testosterone is associated with augmented prostate tissue androgens not otherwise accounted for by peripheral testosterone and results in androgenic stimulation. **A,** Prostate tissue T is significantly increased in the high periprostatic T (DT/PT>1) group (red bars) compared with the low periprostatic T (DT/PT<1) group (blue bars) in the top 2 quartiles. **B, C,** Prostate tissue DHT and AST are both similarly increased in the high periprostatic T group compared with the low periprostatic T group. All statistical comparisons were performed using the Mann-Whitney U test.

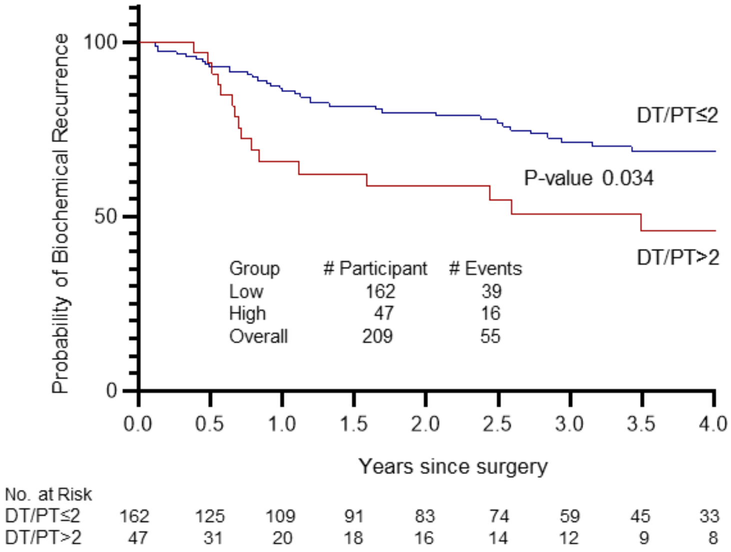

Fig. S3. A DT/PT ratio > 2 is associated with adverse clinical outcomes in men after radical prostatectomy. All 209 men were included in this analysis, whether or not they had PSA persistence. Progression-free survival is defined using PSA recurrence or radiation therapy as clinical events.

Table S1: Patient characteristics and tumor pathology

Characteristic	Overall (N=209)*	DT/PT≤2 (n=162)	DT/PT>2 (n=47)	P**
Age (years), IQR (median)	56-67 (62)	56.2-66.8 (62)	56.5-67 (60)	0.685
Race (White), n (%)	198 (94.7)	155 (95.7)	43 (91.5)	0.272
Pre-op PSA (ng/mL), IQR (median)	4.8- 9.6 (6.2)	4.8- 9.6 (6.2)	4.4- 9.5 (6)	0.664
Prostate weight (g), IQR (median)	36.2-52.2 (42.6)	36.8-51.1 (42.4)	34.4-56.1 (44.7)	0.708
Tumor dimension (mm), IQR (median)	16-25 (21)	15-24 (21)	17-25.8 (21)	0.5
% prostate involved, IQR (median)	10-20 (15)	10-20 (15)	10-20 (15)	0.355
**Pathology, number/cases (%)**				
**Gleason score**				**0.018**
6	12 (5.7)	7 (4.3)	5 (10.6)	
7	168 (80.4)	137 (84.6)	31 (66)	
8-10	29 (13.9)	18 (11.1)	11 (23.4)	
**Final stage**				**0.141**
pT2	102 (48.8)	84 (51.9)	18 (38.3)	
pT3	107 (51.2)	78 (48.1)	29 (61.7)	
ECE	102 (48.8)	75 (46.3)	27 (57.4)	0.238
SVI	18 (8.6)	16 (9.9)	2 (4.3)	0.375
LN (+)	16 (7.7)	13 (8)	3 (6.4)	0.999
Cribriform	63 (30.1)	52 (32.1)	11 (23.4)	0.336

* Four patients had missing values for preop PSA and % prostate involved.
** Mann-Whitney U test for continuous non-normal variables; Chi-squared test or Fisher’s exact test for categorical variables.
ECE: Extracapsular extension; SVI: Seminal vesicle invasion; LN (+): Lymph node positive.

Table S2: Testosterone concentration in peripheral and dorsal venous blood at various DT/PT cut points

	Peripheral (nM)			Dorsal (nM)		
**DT/PT**	Low	High	*P* value	Low	High	*P* value
1	15.24	13.33	0.018	11.63	25.97	<0.001
1.5	14.74	12.99	0.064	12.64	44.1	<0.001
2	14.58	12.71	0.031	13.06	54.17	<0.001
2.5	14.58	12.12	0.002	13.68	64.93	<0.001
3	14.57	12.26	0.005	13.7	81.77	<0.001
**Median in all Patients**		14.25		14.74		0.02

Low: Median concentration below the cut point
High: Median concentration above the cut point

Table S3: Comparison of tissue androgens in the 3rd and 4th quartiles in low versus high groups across a range of DT/PT cut points.

DT/PT Cut point	n above/ n below	T/PT			DHT/PT			3α-AST/PT		
		low	high	*P* value	low	high	*P* value	low	high	*P* value
**1.0**	79/91	0.27	0.42	<0.0001	5.75	7.19	0.0041	5.01	6.28	0.0264
**1.1**	65/105	0.29	0.44	0.0001	6.00	7.43	0.0044	4.90	6.46	0.0157
**1.2**	59/111	0.28	0.50	<0.0001	6.04	7.37	0.0101	5.01	6.28	0.0326
**1.3**	54/116	0.29	0.44	0.0003	6.00	7.37	0.0131	5.02	6.08	0.1200
**1.4**	51/119	0.32	0.42	0.0068	6.14	7.36	0.0576	5.09	5.97	0.2737
**1.5**	51/119	0.32	0.42	0.0068	6.14	7.36	0.0576	5.09	5.97	0.2737
**1.6**	48/122	0.31	0.43	0.0045	6.04	7.36	0.0246	5.02	6.03	0.1350
**1.7**	47/123	0.30	0.44	0.0013	6.04	7.37	0.0155	5.02	6.08	0.0816
**1.8**	45/125	0.31	0.43	0.0041	6.09	7.43	0.0209	5.02	6.18	0.0371
**1.9**	43/127	0.32	0.44	0.0052	6.14	7.36	0.0816	5.02	6.28	0.0513
**2.0**	43/127	0.32	0.44	0.0052	6.14	7.36	0.0816	5.02	6.28	0.0513
**2.1**	39/131	0.31	0.50	0.0007	6.14	7.75	0.0154	5.01	6.64	0.0080
**2.2**	35/135	0.30	0.54	0.0001	6.14	7.82	0.0091	5.01	7.31	0.0029
**2.3**	35/135	0.30	0.54	0.0001	6.14	7.82	0.0091	5.01	7.31	0.0029
**2.4**	33/137	0.30	0.55	<0.0001	6.14	8.19	0.0163	5.00	8.12	0.0010
**2.5**	33/137	0.30	0.55	<0.0001	6.14	8.19	0.0163	5.00	8.12	0.0010
**2.6**	33/137	0.30	0.55	<0.0001	6.14	8.19	0.0163	5.00	8.12	0.0010
**2.7**	33/137	0.30	0.55	<0.0001	6.14	8.19	0.0163	5.00	8.12	0.0010
**2.8**	32/138	0.30	0.55	<0.0001	6.14	8.19	0.0140	5.01	6.97	0.0065
**2.9**	31/139	0.30	0.54	0.0002	6.14	8.56	0.0042	5.01	7.31	0.0029
**3.0**	29/141	0.30	0.53	0.0003	6.14	8.61	0.0062	5.06	6.46	0.0290
